# Chemical Profiles, Anticancer, and Anti-Aging Activities of Essential Oils of *Pluchea dioscoridis* (L.) DC. and *Erigeron bonariensis* L.

**DOI:** 10.3390/plants10040667

**Published:** 2021-03-31

**Authors:** Abdelbaset M. Elgamal, Rania F. Ahmed, Ahmed M. Abd-ElGawad, Abd El-Nasser G. El Gendy, Abdelsamed I. Elshamy, Mahmoud I. Nassar

**Affiliations:** 1 Department of Chemistry of Microbial and Natural Products, National Research Centre, 33 El-Bohouth St., Dokki, Giza 12622, Egypt; 2 Chemistry of Natural Compounds Department, National Research Center, 33 El Bohouth St., Dokki, Giza 12622, Egypt; rf.ali@nrc.sci.eg (R.F.A.); mnassar_eg@yahoo.com (M.I.N.); 3 Department of Botany, Faculty of Science, Mansoura University, Mansoura 35516, Egypt; aibrahim2@ksu.edu.sa; 4 Medicinal and Aromatic Plants Research Department, National Research Centre, 33 El Bohouth St., Dokki, Giza 12622, Egypt; ag.el-gendy@nrc.sci.eg

**Keywords:** horseweed, wavy-leaf fleabane, sesquiterpenes, cytotoxicity, anti-senility

## Abstract

Plants belonging to the Asteraceae family are widely used as traditional medicinal herbs around the world for the treatment of numerous diseases. In this work, the chemical profiles of essential oils (EOs) of the above-ground parts of *Pluchea dioscoridis* (L.) DC. and *Erigeron bonariensis* (L.) were studied in addition to their cytotoxic and anti-aging activities. The extracted EOs from the two plants via hydrodistillation were analyzed by gas chromatography-mass spectroscopy (GC-MS). GC-MS of EO of *P. dioscoridis* revealed the identification of 29 compounds representing 96.91% of the total oil. While 35 compounds were characterized from EO of *E. bonariensis* representing 98.21%. The terpenoids were found the main constituents of both plants with a relative concentration of 93.59% and 97.66%, respectively, including mainly sesquiterpenes (93.40% and 81.06%). *α*-Maaliene (18.84%), berkheyaradulen (13.99%), dehydro-cyclolongifolene oxide (10.35%), aromadendrene oxide-2 (8.81%), *β*-muurolene (8.09%), and *α*-eudesmol (6.79%), represented the preponderance compounds of EO of *P. dioscoridis*. While, trans-α-farnesene (25.03%), *O*-ocimene (12.58%), isolongifolene-5-ol (5.53%), α-maaliene (6.64%), berkheyaradulen (4.82%), and *α*-muurolene (3.99%), represented the major compounds EO of *E. bonariensis*. A comparative study of our results with the previously described data was constructed based upon principal component analysis (PCA) and agglomerative hierarchical clustering (AHC), where the results revealed a substantial variation of the present studied species than other reported ecospecies. EO of *P. dioscoridis* exhibited significant cytotoxicity against the two cancer cells, MCF-7 and A-549 with IC_50_ of 37.3 and 22.3 μM, respectively. While the EO of the *E. bonariensis* showed strong cytotoxicity against HepG2 with IC_50_ of 25.6 μM. The EOs of *P. dioscoridis*, *E. bonariensis*, and their mixture (1:1) exhibited significant inhibitory activity of the collagenase, elastase, hyaluronidase, and tyrosinase comparing with epigallocatechin gallate (EGCG) as a reference. The results of anti-aging showed that the activity of mixture (1:1) > *P. dioscoridis* > *E. bonariensis* against the four enzymes.

## 1. Introduction

Natural products derived from the plant kingdom represented potent resources for foods, cosmetics, and traditional medicines [1,2]. Many scientists focused on the study of the chemical characterization of essential oils (EOs) along with their pharmaceutical effects for many decades [3,4,5,6]. Due to the complicated composition from different isoprenoids based compounds [7], EOs exhibited several significant biological effects including anti-inflammatory, antipyretic [8], antioxidant [9,10], allelopathy [8,11,12,13,14,15,16,17], antiulcer [18], antimicrobial [8,19], and hepatoprotective [20]. EOs have been reported as potent agents against degenerative diseases via inhibition of oxidative stress due to the strong free radical scavenging activity [21].

Plants belonging to *Conyza* genus (Family Asteraceae), including around 150 plant species [22], were described as important traditional medicinal plants in the treatment of toothache, skin diseases, rheumatism, haemorrhoidal, diarrhoeal, and injuries bleeding [23,24]. Some members of Asteraceae were named *Conyza* formerly; however, some taxa names have been changed later based on taxonomic criteria. From these taxa, *Conyza dioscoridis* (L.) Desf. that its accepted name now is *Pluchea dioscoridis* (L.) DC., and *Conyza linifolia* (Willd.) Täckh. that now have been accepted as *Erigeron bonariensis* L. [25,26]. Studying various criteria of the plant species such as morphological, anatomical, molecular, and chemical properties, providing valuable information for taxonomists, thereby, some taxa names have been changed [27,28]. EOs analysis has been reported to provide profitable information for chemotaxonomy [27,29].

*Pluchea dioscoridis* (L.) DC. (syn. *Conyza dioscoridis* (L.) Desf.) is a widely distributed wild plant in the Nile delta, Mediterranean coast, Sinai Peninsula, Western Desert, and Eastern Desert [25]. This plant was described in folk medicines for the treatment of some diseases as ulcer, colic, carminative, epilepsy in children, rheumatic pains, and cold [30]. Many documents described that the different extracts of this plant have several potent biological activities comprising anti-inflammation, antiulcer, antidiabetic, antinociceptive, antipyretic, ant-diarrheal, antibacterial, antifungal, and free radical scavenging activities, along with diuretic effect [30,31,32,33]. Many metabolites were isolated and characterized from *P. dioscoridis* including steroids, triterpenes [30], flavonoids, and phenolic acids [30,32].

The chemical constituents of EO of *E. bonariensis* collected from Alexandria, Egypt has been reported in addition to its antimicrobial and insecticidal activities [34]. In this study, 25 compounds were identified from EO of *E. bonariensis* including sesqui- and monoterpenes. From the total of this oil, α-bergamotene, and D-limonene represented the mains with concentrations of 27.4 and 22.5%. In the same report, EO of *C. linifolia* was documented to exhibit antibacterial potentiality against *B. subtilis* with MIC of 125 mg/mL [34,35]. Little reports concerning the chemical profiles as well as biological activities of *Erigeron bonariensis* L. (syn. *Conyza linifolia* (Willd.) Täckh.) have been recorded.

We hypothesized that these two plant species were formerly named *Conyza*, and their names were changed. Therefore, the chemical characterization of their EOs could be valuable in their chemotaxonomy. Herein, this work aimed to (i) identify the chemical profiles of EOs from *P. dioscoridis* and *E. bonariensis*, collected from Egypt, (ii) establish comparative profiles of the two plants based upon chemometric analysis with other reported ecospecies, (iii) study the cytotoxic activity of the EOs of the two plants against several human cancer cell lines, and (iv) assess in vitro anti-aging potentialities of the EOs of the two plants.

## 2. Results and Discussion

### 2.1. Chemical Compositions of EO of P. dioscoridis

**Figure 1:**
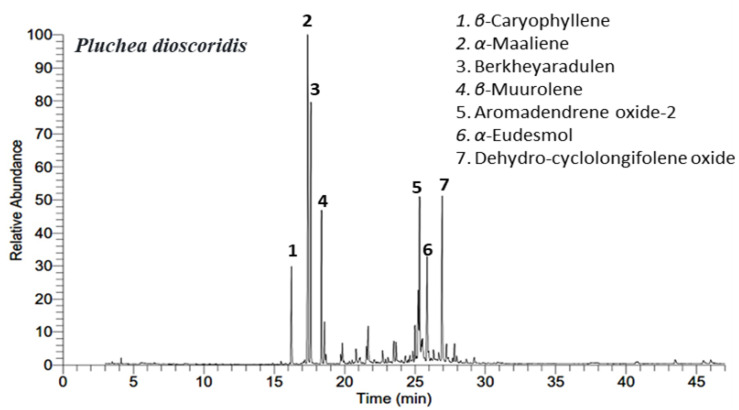
Gas chromatography-mass spectroscopy (GC-MS) chromatograme of the essential oils (EO) of *Pluchea dioscoridis*. The main peaks were numbered (1–7).

The chemical characterization of *P. dioscoridis* EO was extracted via hydro-distillation afforded golden yellow (0.037%). The chemical profiles of the extracted EO were assigned depending upon the GC-MS analysis. The GC-MS chromatogram of the EO is presented in Figure 1 exhibiting the main peaks from all identified components. Twenty-nine compounds were identified from the EO of *P. dioscoridis* represented 96.91% of the total oil. All the identified compounds along with their chemical and physical properties were summarized in Table 1.

The constituents of EO of *P. dioscoridis* were characterized by the presence of four classes of compounds including sesqui- (93.40%), and monoterpenes (0.19%), carotenoid derived compounds (0.28%) in addition to other acyclic compounds (1.33%). The terpenoids were found as abundant compounds with a relative concentration of 93.59% in addition to traces of carotenoids and acyclic compounds with a complete absence of diterpenoids. GC-MS analysis of EO derived from *E. bonariensis*, revealed the presence of four categories of compounds comprising sesqui- (81.06%), and monoterpenes (14.16%), diterpenes (2.44%) in addition to other acyclic compounds (0.55%). Furthermore, the terpenoids were characterized as the main components by a relative concentration of 95.22% with traces of diterpenoids and other compounds. These results deduced the fact of the preponderance of the terpenoids in the different species of *Conyza* genus [32,34,37].

Sesquiterpenoids were found as the main compounds of the EO of *P. dioscoridis* with mixtures of oxygenated and non-oxygenated compounds. The abundance of sesquiterpenes was found in full agreement with previous data of EOs of this plant [32,38]. From all identified sesquiterpenes, α-maaliene (18.84%), berkheyaradulen (13.99%), dehydro-cyclolongifolene oxide (10.35%), aromadendrene oxide-2 (8.81%), *β*-muurolene (8.09%), α-eudesmol (6.79%), *β*-caryophyllene (4.95%), t-muurolol (3.88%), represented the major compounds.

Berkheyaradulen, muurolene, eudesmol, tau-muurolol, and caryophyllene, were found as marker compounds for this plant in the previous study [32] and this data is in the same line with our results. While the reported data of EO of the leaves of this plant [38] exhibited variations in chemical constituents than those data described previously by our team [32] and also than our results herein. Elshamy, et al. [32] documented that *α*-cadinol is the main sesquiterpene and this data is different than our results in which *γ*-cadinol is present as a minor compound. Additionally, eudesmol and tau-muurolol were reported as major sesquiterpenes in EO of the leaves of this plant, and this data agreed with our results.

The results of GC-MS of EO of *P. dioscoridis* revealed that the monoterpenes are traces with only one compound, α-pinene (0.19%). The scarcity of monoterpenes is consistent with the results of Elshamy, et al. [32] and El-Seedi, et al. [38].

In EO derived from *P. dioscoridis*, diterpenes were completely absent and this result is inconsistent with the published data [32,37], while El-Seedi, et al. [38] characterized only one diterpene, phytol, from the leaves of this plant. *α*-ionone was the only identified carotenoid-derived compound from EO of *P. dioscoridis* that was not reported before from this plant [32].

The other compounds (1.33%) including hydrocarbons were characterized as traces in EO of *P. dioscoridis* that was in agreement with the previous data [32,39]. In contrast, El-Seedi, et al. [38] documented that the monoterpenoid compounds represented a high concentration (26.6%) of the total mass of EO of the leaves of *P. dioscoridis*.

### 2.2. Chemical Compositions of EO of E. bonariensis

**Figure 2:**
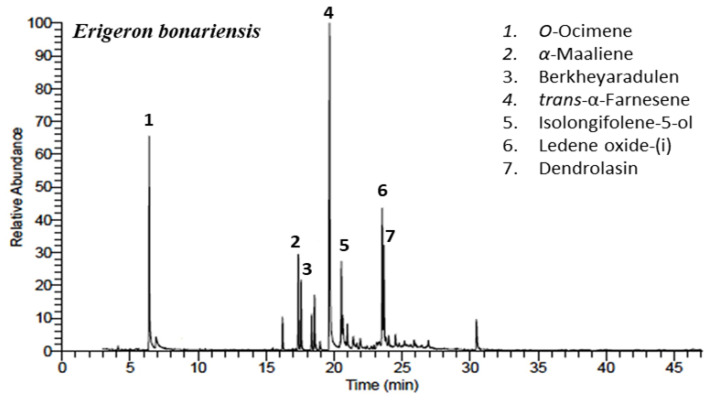
GC-MS chromatogrames of the EO of *Erigeron bonariensis*. The main peaks were numbered (1–7).

The hydro-distillation of the above-ground parts of *E. bonariensis* afforded golden yellow EO (0.049%). The chemical characterization of the extracted EO was performed based on the GC-MS analysis. Figure 2 represented the GC-MS chromatogram including the major peaks. Thirty-five components were assigned representing 98.21% of the total oil mass. The characterized constituents as well as retentions times (RIs), molecular formulas (MFs), and literature and calculated Kovats indexes (KIs) were compacted in Table 1.

In EO of *E. bonariensis,* sesquiterpenes represented also the main constituents including several oxygenated and non-oxygenated metabolites. With a relative concentration of 81.06% of sesquiterpenes, our results are completely agreed with the previous data by Harraz, et al. [34] that reported a relative concentration of 92.50%; trans-α-Farnesene (25.03%), isolongifolene-5-ol (5.53%), *α*-maaliene (6.64%), berkheyaradulen (4.82%), and *α*-muurolene (3.99%) were found the main sesquiterpenoid contents. The main sesquiterpene, trans-α-farnesene, was widely distributed in the EOs of *Conyza* species such as *C. bonariensis* (*≈E. bonariensis*) collected from Venezuela and Vietnam [40], *C. canadensis* [39], and *C. sumatrensis* [41]. However, in the only stated study of EO of *E. bonariensis* [34], α-bergamotene was described as the main compound in addition to some farnesene derivatives such as, *β*-farnesene, and (*E*)-farnesene epoxide. The abundance of α-maaliene (6.64%), berkheyaradulen (4.82%), and α-muurolene (3.99%) were found in perfect harmony with our results of EO of *P. dioscoridis*. The variations of secondary metabolites comprising EOs might be attributed to the plant age and development, plant organs, as well as the environmental factors including such as altitude, seasonality, atmospheric composition and temperature, and water availability [11,42,43].

Monoterpenes represented a remarkable concentration of the EO of *E. bonariensis* with a wealth of *O*-ocimene (12.58%). *O*-Ocimene was reported here for the first time in EO of this plant, contrariwise, Harraz, et al. [34] reported the complete absence of it from EO of the aerial parts of this plant collected from Alexandria, Egypt.

The diterpenoids were represented by a relative concentration of 2.44% from over all mass of the oil of *E. bonariensis*. The total relative concentration of diterpenes was determined in the EO of *E. bonariensis* with only one compound, neophytadiene, which is not reported before from the EO of this plant [34].

Carotenoid derived compounds were not identified from the EO of *E. bonariensis* and this result is in harmony with Mabrouk, et al. [37]; also, hydrocarbons and the other components were represented by traces in EO of *E. bonariensis* (0.55%) that agreed the previous described studies [32,39].

### 2.3. Chemometric Analysis

**Figure 3:**
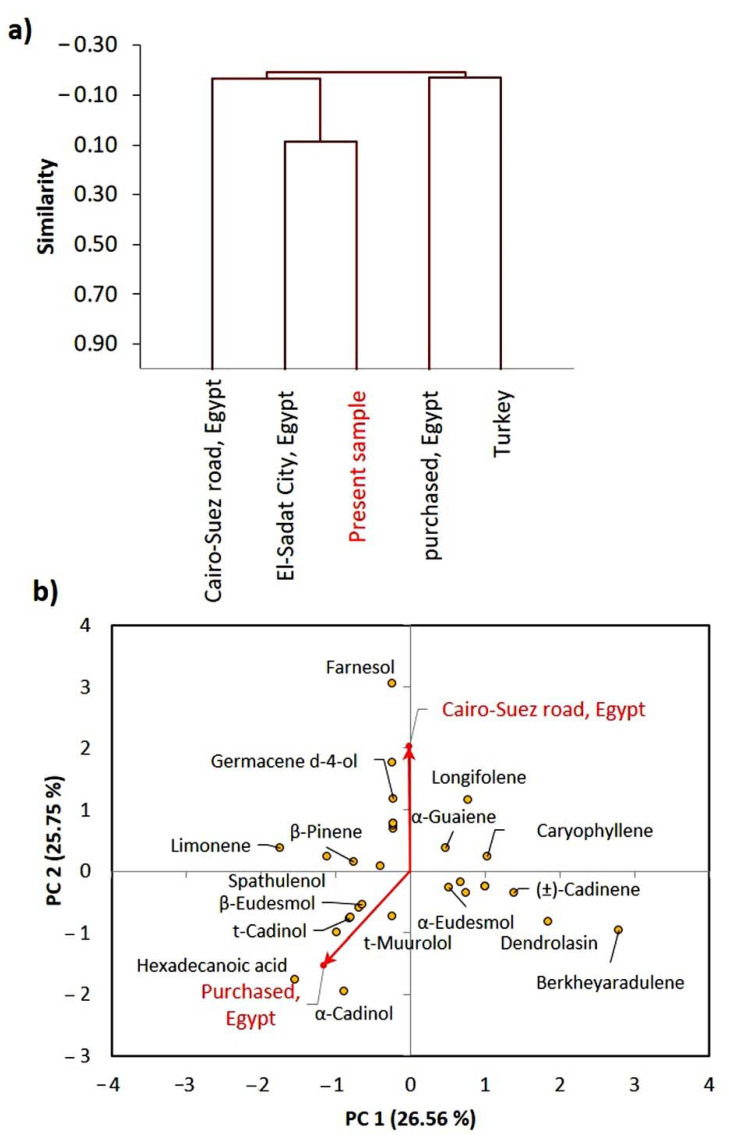
Chemometric analysis of the EOs from the present studied *Pluchea dioscoridis* ecospecies and other reported ecospecies. (**a**) agglomerative hierarchical clustering (AHC) and (**b**) principal component analysis (PCA).

The EOs chemical compositions of the major compounds (>3%), reported from different ecospecies of *P. dioscoridis* and *E. bonariensis* were constructed in a matrix. These collected data were subjected to agglomerative hierarchical clustering (AHC) and principal component analysis (PCA). The cluster analysis of *P. dioscoridis* EOs showed that the present studied sample of *P. dioscoridis* is closely correlated to the Egyptian ecospecies collected from El-Sadat City, and little correlated to that collected from Cairo–Suez desert road, Egypt (Figure 3a). However, the present sample was different than those purchased from a commercial source in Cairo, Egypt. This means that the commercial samples are not in pure form or may be mixed with other plants.

The PCA of the *P. dioscoridis* ecospecies showed that the sample collected from Cairo-Suez desert road, Egypt is mainly characterized by farnesol, germacene d-4-ol, and longifolene (Figure 3b). However, the purchased sample from a commercial source in Cairo, Egypt is characterized by hexadecanoic acid and *α*-cadinol.

**Figure 4:**
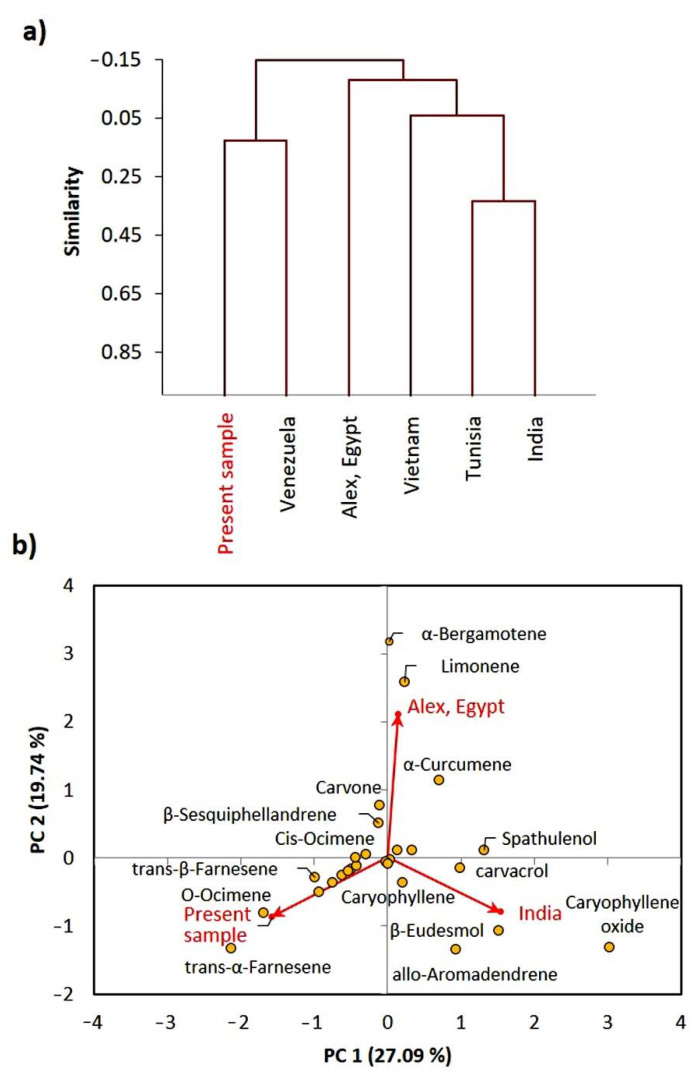
Chemometric analysis of the EOs from the present studied *Erigeron bonariensis* ecospecies and other reported ecospecies. (**a**) agglomerative hierarchical clustering (AHC) and (**b**) principal component analysis (PCA).

On the other side, the cluster analysis of *E. bonariensis* EOs revealed that the present Egyptian sample is closely related to the Venezuelan ecospecies, while it was different than other ecospecies (Figure 4a).

While the Indian and Tunisian ecospecies showed a close relation in the composition of the EO. The PCA showed that the present sample of *E. bonariensis* is characterized by *trans-α*-Farnesene, O-ocimene, and *trans-β*-Farnesene (Figure 4b). The sample collected from Alexandria, Egypt, showed a close correlation with *α*-bergamotene, limonene, and *α*-curcumene, while the Indian ecospecies is characterized by *β*-eudesmol, caryophyllene oxide, allo-aromadendrene, and carvacrol.

The observed variation among the present samples and other reported ones revealed the profitable information derived from the EOs analysis, which could be a useful tool in chemotaxonomy [27].

### 2.4. Anti-Aging Activity 

**Figure 5:**
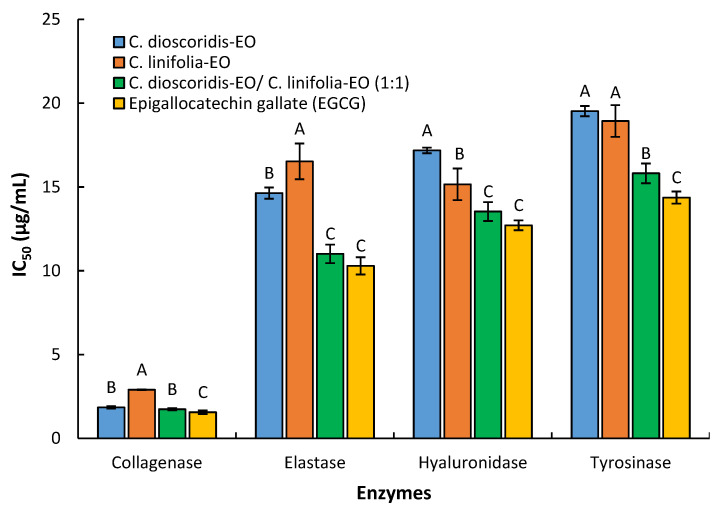
Anti-aging activities of the EOs extracted from *Pluchea dioscoridis* and *Erigeron bonariensis* against the four enzymes: collagenase, elastase, hyaluronidase, and tyrosinase. Values are IC50 (µg/mL) as an average of three replicates and the bars representing the standard deviation. Different letters (A, B, and C) within each enzyme mean values significant at 0.05 probability level after Duncan’s test.

The EOs from *P. dioscoridis*, *E. bonariensis*, and the mixture of the two EOs (1:1) have a strong inhibitory activity of the collagenase, elastase, hyaluronidase, and tyrosinase (Figure 5). All the EO treatments exhibited potent inhibition of collagenase enzyme with IC_50_ of 1.85, 2.90, and 1.73 µg/mL for *P. dioscoridis*, *E. bonariensis,* and the mixture, respectively. Furthermore, the three EO treatments strongly inhibit the elastase enzyme with respective values of IC_50_ of 14.63, 16.52, and 11.01 µg/mL. Furthermore, strong suppression of hyaluronidase was demonstrated via the three EO treatments based upon the respective observed values of IC_50_ of 17.18, 15.16, and 13.54 µg/mL. By the same, the three tested displayed strong tyrosinase enzyme inhibition with IC_50_ values at 19.52, 18.93, and 15.81 µg/mL, respectively. All the results were constructed based upon comparing with the polyphenolic compound, epigallocatechin gallate (EGCG), as a standard ant-aging reference [44] that exhibit inhibition of collagenase, elastase, hyaluronidase, and tyrosinase with IC_50_ of 1.56, 10.29, 12.71, and 14.37 µg/mL.

In the matrix of extracellular, the elastin and hyaluronan degradation were principally correlated with the two respective proteolytic enzymes, elastase, and hyaluronidase that cause the main reasons for aging of the skin such as wrinkles, sagging. Moreover, tyrosinase caused the regulation of the synthesis of melanin in human melanocytes that lead to skin ailments.

In the present study, the anti-aging of the 1:1 mixture of the two EOs was evaluated to study the synergetic effects of the combination of the two EOs. Results revealed that the EO of *P. dioscoridis*, *E. bonariensis,* and the mixture of the two EOs (1:1) have strong anti-aging activity. These results might be attributed to the chemical components of these oils. The anti-aging activity was directly correlated with antioxidant potentiality [45]. The main constituents in both EOs, sesquiterpenes, were described to play a significant role as antioxidants, anti-inflammatory agents, and thus anti-aging [45]. Tu and Tawata [45] reported that EO of the leaves of *Alpinia zerumbet* exhibit antioxidant and anti-aging activities due to the high concentration of terpenoids, especially sesquiterpenes. Furthermore, the monoterpenes were documented as active anti-aging agents in EO of *Juniperus communis* [46] and *Origanum vulgare* [47]. These reports concluded that the increase of free radical scavenging constituents in EOs lead to an increase in their anti-aging activity. Based upon this fact, the high concentrations of terpenes especially the oxygenated sesqui- and monoterpenes caused increasing in the anti-aging activity of EOs of these two plants. All these reported data deduced the role of synergetic effects between the components of the EOs. This fact of the role of synergetic effect was very clear in our results in which the mixture of the two EOs (1:1) exhibited better activity than the individual EO of each plant. In the mixture of the two EOs, the raising of concentration of oxygenated terpenes as well the synergetic effects between the components caused increasing of the inhibition potentiality. 

### 2.5. Cytotoxic Activity of EOs of P. dioscoridis and E. bonariensis

**Figure 6:**
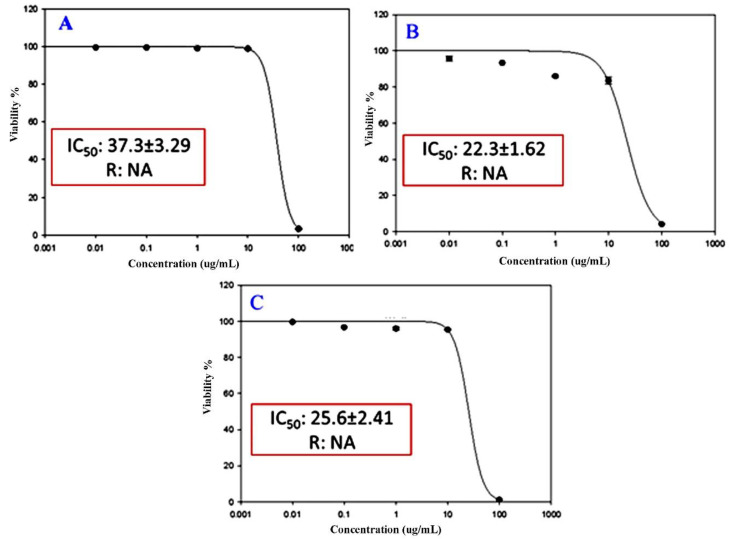
Cytotoxicity of EOs of (**A**) *Pluchea dioscoridis* against MCF-7 cells, (**B**) *P. dioscoridis* against A-549 cells, and (**C**) *Erigeron bonariensis* against HepG2.

The cytotoxicity of EOs of the above-ground parts of the two plants, *P. dioscoridis* and *E. bonariensis*, as well as a mixture of the two EOs (1:1) against the three cancer cell lines, breast adenocarcinoma cells (MCF-7), lung cancer cells (A-549), and hepatocellular carcinoma cells (HepG2) are shown in Figure 6. The results exhibited that the EO of *P. dioscoridis* have a significant inhibition of the two cancer cells, MCF-7 and A-549, with IC_50_ of 37.3 and 22.3 μM, respectively (Figure 6A,B), without any activity against HepG2. While, the EO of the *E. bonariensis* showed inhibitory potentiality only against HepG2 with IC_50_ of 25.6 μM (Figure 6C), with negative results against MCF-7 and A-549. The 1:1 mixture of the two EOs did not exhibit any activity against the three cancer cells. 

The significant activities of the two EOs might be attributed to the chemical composition in which the synergetic effect of the compounds contributes to this activity [48]. The sesquiterpenes in both forms, oxygenated and hydrocarbons, represented very effective compounds as anticancer leaders [49,50]. Several reports deduced that the increasing of sesquiterpene contents in EOs caused increasing in anticancer activity [51,52]. For example, caryophyllene with high concentration in EOs was reported as a known potential cytotoxic agent especially against the growth of breast adenocarcinoma cells (MCF-7) [53,54].

The present data revealed that these two EOs are selective against the tested cancer cells. This selectivity was in full agreement with several documented results of EOs derived from other plants. For example, EO derived from *Sideritis perfoliata*, *Satureia thymbra*, *Salvia officinalis*, *Laurus nobilis,* and *Pistacia palestina* were found to have selective inhibitory effects against, amelanotic melanoma (C32), renal celladenocarcinoma (ACHN), hormone-dependent prostatecarcinoma (LNCaP), and breast cancer (MCF-7) [55]. Moreover, EOs extracted from the three plants, *Satureja montana*, *Coriandrum sativum,* and *Ocimum basilicum*, were found to have selective cytotoxic activity against HeLa, MDA-MB-453, K562, and MRC-5 [56]. The disappearance of the mixtures of the two EOs (1:1) might be ascribed to the negative synergetic effects of each EO upon the other and this phenomenon was reported in some reports. Haroun and Al-Kayali [57] found that the different extracts of *Thymbra spicata* showed positive synergetic effects via combination with some references antibiotics against some strains of bacteria and a while negative synergetic effects against other strains.

## 3. Materials and Methods

### 3.1. Plant Materials Collection and Preparation

The above-ground parts of *P. dioscoridis* and *E. bonariensis*, were collected from two populations along the Cairo–Alexandria desert road, Egypt in November 2019. From each population, the healthy and fresh plant samples were clipped from three individuals and pooled as composite samples (two per each plant; *P. dioscoridis* and *E. bonariensis*). The two plants were authenticated according to Tackholm [58] and Boulos [25]. Voucher specimens (CZ-D-x908-019 & CZ-L-x909-019) have been deposited in the herbarium of the National Research Center, Egypt. The above-ground parts were dried in the shade, ground into a fine powder, and packed in paper bags till further analysis [13].

### 3.2. Extraction of EOs

The air-dried powder of the above-ground parts of the *P. dioscoridis*, and *E. bonariensis*, (200 gm, each) were subjected separately to Clevenger-type apparatuses using round flask (2.5 L) comtaining water (1.5 L) for hydro-distillation for 3 h. The oily layer of each plant was isolated separately by *n*-hexane, then dried using anhydrous Na_2_SO_4_ (0.5 g), and finally stored in glass vials in the freezer till further analysis via GC-MS. This extraction of the EO of each plant was repeated as duplicates. 

### 3.3. Gas Chromatography-Mass Spectroscopy (GC-MS) Analysis and Chemical Components Investigations 

The four EOs samples (two samples for each plant) were analyzed via Gas Chromatography-Mass Spectroscopy (GC-MS) at National Research Center, Egypt [8]. The adjustment of the GC/MS instrument specifications has occurred as the following conditions: TRACE GC Ultra Gas Chromatographs (THERMO Scientific™ Corporate, Waltham, MA, USA), lined with a Thermo Scientific ISQ™ EC single quadrupole mass spectrometer. The GC-MS system was equipped with a TR-5 MS column with a dimension of 30 m × 0.32 mm i.d., 0.25 μm film thickness. Helium as carrier gas at a flow rate of 1.0 mL/min with a split ratio of 1:10 using the following temperature program: 60 °C for 1 min; rising at 4.0 °C/min to 240 °C and held for 1 min was used for the analyses. Both injector and detector were held at 210 °C. An aliquot of 1 μL of diluted samples in hexane (1:10, *v/v*) was always injected. Mass spectra were recorded by electron ionization (EI) at 70 eV, using a spectral range of *m/z* 40–450.

Chemical constituent of the EOs under investigations was characterized by Automated Mass spectral Deconvolution and Identification (AMDIS) software (www.amdis.net, accessed on 2 January 2020), retention indexes (relative to *n*-alkanes C_8_-C_22_), comparison of the mass spectrum with authentics (if available), and Wiley spectral library collection and NSIT library database (Gaithersburg, MD, USA; Wiley, Hoboken, NJ, USA).

### 3.4. Anti-Aging Activity of the EOs

#### 3.4.1. Anti-Collagenase Assay

The anti-collagenase assay of the two studied plants EOs as well as the 1:1 mixture were performed according to Thring, et al. [59] with minor modifications for use in a microplate reader. The assay was performed in 50 mM tricine buffer (pH 7.5) with 400 mM NaCl and 10 mM CaCl_2_. Collagenase from *Clostridium histolyticum* (ChC–EC.3.4.23.3) was dissolved in a buffer for use at an initial concentration of 0.8 units/mL according to the supplier’s activity data. The synthetic substrate *N*-[3-(2-furyl) acryloyl]-Leu-Gly-Pro-Ala (FALGPA) was dissolved in tricine buffer to 2 mM. Two studied EOs and the mixture of EOs of the two plants (1:1, *w*/*w*), separately, were incubated with the enzyme in a buffer for 15 min before adding substrate to start the reaction. Absorbance at 490 nm was measured using a Microplate reader (TECAN, Group Ltd., Männedorf, Switzerland). Epigallocatechin gallate (EGCG) was used as a positive control.

#### 3.4.2. Anti-Elastase Assay

For anti-elastase inhibitory assay the two studied plants EOs as well as the 1:1 mixture, this assay was performed according to Kim, et al. [60] with minor modifications. Briefly; Porcine pancreatic elastase, was dissolved to make a 3.33 mg/mL stock solution in sterile water. The substrate, N-succinyl-Ala-Ala-Ala-*p*-nitroanilide (AAAPVN) was dissolved in buffer at 1.6 mM. The test EOs were incubated with the enzyme for 15 min before adding substrate to begin the reaction. The final reaction mixture (250 μL total volume) contained buffer, 0.8 mM AAAPVN, 1 μg/mL PE and 25 μg test sample. The studied EOs and a mixture of EOs of the two plants (1:1, *w*/*w*), separately, were incubated. EGCG was used as a positive control. Absorbance values at 400 nm were measured in 96 well microtitre plates using a Microplate reader (TECAN, Inc.). The percentage inhibition for this assay is calculated.

#### 3.4.3. Anti-Tyrosinase Assay

Assays of tyrosinase inhibition of the two plants EOs, as well as the 1:1 mixture, were carried out via measuring of L-DOPA chrome formation according to the described protocol of Batubara, et al. [61]. Briefly, the two EOs and a mixture of them (1:1, *w*/*w*), separately, were dissolved in a solvent with three certain concentrations (10, 100, and 250 μg/mL). The assays were performed by insertion of the following components: (a) phosphate buffer (120 μL, 20 mM, pH 6.8), (b) 20 μL sample, and (c) 20 μL mushroom tyrosinase (500 U/mL in 20 mM phosphate buffer) in 96-well plates. After 15 min of incubation at 25 °C, the intiation of reaction was occurred by insertion of 20 μL L-tyrosine solution (0.85 mM) for every well and followed by incubation for 10 min at room temperature. The activity of the enzyme was monitored at 475 nm using a Microplate reader (TECAN, Inc.). EGCG was used as a positive control. The calculation of the tyrosinase inhibition % was performed via the following equation:

Tyrosinase inhibition (%) = [(A − B) − (C − D)]/(A−B) × 100(1)

where A is the absorbance of the control with the enzyme, B is the absorbance of the control without the enzyme, C is the absorbance of the test sample with the enzyme, and D is the absorbance of the test sample without the enzyme.

#### 3.4.4. Anti-Hyaluronidase Assay

The fluorimetric Morgan–Elson assay method was performed according to Reissig, et al. [62] that modified by Takahashi, et al. [63]. In a brief description, a 5 μL of tested EOs and a mixture of EOs of the two plants (1:1, *w*/*w*), separately, were incubated for 10 min at 37 °C with bovine hyaluronidase (1.50 U) in 100 μL of 20 mM sodium phosphate buffer solution (pH 7.0), sodium chloride (77 mM), in addition to 0.01% bovine serum albumin (BSA). The assay reaction was initiated via adding the hyaluronic acid sodium salt (100 μL) from rooster comb (0.03% in 300 mM sodium phosphate, pH 5.35) to the incubation mixture, then the mixture was incubated at 37 °C for 45 min. The precipitation of undigested hyaluronic acid was carried out by 1 mL acidic solution of albumin, involving 0.1% BSA in sodium acetate (24 mM) and acetic acid (79 mM, pH 3.75). The mixture was stoped by allowing it for 10 min at room temperature, and fluorescence was detected using a Tecan Infinite microplate reader at 545 nm excitation and 612 nm emission EGCG was used as a positive control.

The percentage of the collagenase, elastase, and hyaluronidase inhibition was calculated via the following equation:

Enzyme inhibition (%) = [1 − (*S*/*C*) × 100] (2)

where *S*: the corrected absorbance of the samples containing elastase inhibitor (the enzyme activity in the presence of the samples); and *C*: the corrected absorbance of controls (the enzyme activity in the absence of the samples).

The IC_50_, the concentration required to inhibit 50% of the enzyme under the assay conditions, was estimated from graphic plots of the dose-response curve for each concentration using Graphpad Prism software (San Diego, CA, USA).

### 3.5. Cytotoxicity of the Two EOs

Cytotoxic activity of the *P. dioscoridis* and *E. bonariensis* EOs and a mixture of them (1:1, *w*/*w*), separately were carried out against the three human cancer cells, breast adenocarcinoma cells (MCF-7), lung cancer cells (A-549), and hepatocellular carcinoma cells (HepG2), using sulforhodamine B (SRB) protocol.

#### 3.5.1. Cell Culture

The three cancer cell lines, breast adenocarcinoma cells (MCF-7), lung cancer cells (A-549), and hepatocellular carcinoma cells (HepG2) were obtained from VACCERA, Mokatam, Giza, Egypt. Cells were maintained in DMEM media supplemented with 100 mg/mL of streptomycin, 100 units/mL of penicillin, and 10% of heat-inactivated fetal bovine serum in humidified, 5% (*v/v*) CO_2_ atmosphere at 37 °C.

#### 3.5.2. Cytotoxicity Assay

Cell viability was assessed by SRB assay. Aliquots of 100 μL cell suspension (5 × 10^3^ cells) were in 96-well plates and incubated in complete media for 24 h. Cells were treated with another aliquot of 100 μL media containing EOs and a mixture of them (1:1, *w*/*w*), separately, at various concentrations ranging from (0.01, 0.1, 1, 10, and 100 ug/mL). After 72 h of drug exposure, cells were fixed by replacing media with 150 μL of 10% TCA and incubated at 4 °C for 1 h. The TCA solution was removed, and the cells were washed 5 times with distilled water. Aliquots of 70 μL SRB solution (0.4% *w/v*) were added and incubated in a dark place at room temperature for 10 min. Plates were washed 3 times with 1% acetic acid and allowed to air-dry overnight. Then, 150 μL of TRIS (10 mM) was added to dissolve protein-bound SRB stain; the absorbance was measured at 540 nm using a BMG LABTECH®- FLUOstar Omega microplate reader (Ortenberg, Germany) [64,65].

### 3.6. Data Treatment

The data of the anti-aging activity of various enzymes were presented in three replications and subjected to one-way ANOVA followed by Duncan’s test using CoStat version 6.311 (CoHort, Monterey, CA, USA, http://www.cohort.com).

A matrix of the concentration of a total of 30 major chemical compounds (>3%) identified in the EO of five *P. dioscoridis* ecospecies was constructed, these samples were (1) present sample; (2) purchased from a market in Cairo, Egypt; (3) collected from El-Sadat City, Egypt; (4) collected from Cairo-Suez desert road, Egypt dring April; and (5) collected from Turkey. While for *E. bonariensis*, a matrix of 27 major chemical compounds (>3%) represented six samples was designed, these samples were (1) present sample; (2) collected from Alexandria, Egypt; (3) collected from Monastir, Tunisia; (4) collected from Venezuela; (5) collected from Vietnam; and (6) collected from India. The matrices were subjected to principal component analysis (PCA) and agglomerative hierarchical clustering (AHC) via XLSTAT statistical computer software package (version 2018, Addinsoft Inc., New York, NY, USA).

## 4. Conclusions

Herein, the GC-MS analysis of EOs of the above-ground parts of *P. dioscoridis* and *E. bonariensis*, revealed the identification of 29 and 35 compounds, respectively. Sesquiterpenes were characterized as the main components of EOs derived from the two plants. The major components of EO of *P. dioscoridis* were *α*-maaliene, berkheyaradulen, dehydro-cyclolongifolene oxide, aromadendrene oxide-2, and *β*-muurolene. While, *trans*-α-farnesene, *O*-ocimene, and α-maaliene represented the abundant constituents of *E. bonariensis* EO. The observed variation in the EOs composition among the studied ecospecies and that reported support the changing of the taxa names. EO of *P. dioscoridis* exhibited cytotoxicity against the two cancer cells, MCF-7 and A-549, while the EO of the *E. bonariensis* showed activity only against HepG2. The EOs of *P. dioscoridis* and *E. bonariensis* as well as the mixture of them (1:1), exhibited significant anti-aging activity in which the mixture (1:1) > *P. dioscoridis* > *E. bonariensis*. All these data deduced the studied EOs of these two plants may be used as antiaging and anticancer leading agents.

## Figures and Tables

**Table 1:** Components of essential oils of *Pluchea dioscoridis* and *Erigeron bonariensis*.

No	Rt ^[a]^	Compound Name	MF	KI_Lit_^[b]^	KI_Exp_^[c]^	Relative Concentration %	
						*P. discodirdis*	*E. bonariensis*
		**Monoterpenes**				**0.19%**	**14.16%**
1	4.13	*α*-Pinene	C_10_H_16_	933	934	0.19 ± 0.01	0.18 ± 0.01
2	5.43	*α*-Myrcene	C_10_H_16_	991	990	------	0.20 ± 0.01
3	6.41	*O*-Ocimene	C_10_H_16_	1012	1012	------	12.58 ± 0.09
4	6.91	D-Limonene	C_10_H_16_	1035	1036	------	1.20 ± 0.04
		**Sesquiterpenes**				**93.4%**	**81.06%**
5	16.22	*β*-Caryophyllene	C_15_H_24_	1418	1420	4.95 ± 0.07	2.17 ± 0.04
6	16.96	Aromandendrene	C_15_H_24_	1439	1448	------	0.12 ± 0.03
7	17.17	*α*-Guaiene	C_15_H_24_	1439	1439	0.21 ± 0.02	0.11 ± 0.02
8	17.39	*α*-Maaliene	C_15_H_24_	1480	1479	18.84 ± 0.08	6.64 ± 0.09
9	17.60	Berkheyaradulen	C_15_H_24_	1492	1493	13.99 ± 0.09	4.82 ± 0.06
10	18.36	*β*-Muurolene	C_15_H_24_	1493	1493	8.09 ± 0.05	2.37 ± 0.04
11	18.57	*α*-Muurolene	C_15_H_24_	1498	1499	2.20 ± 0.04	3.99 ± 0.07
12	18.98	Bicyclogermacrene	C_15_H_24_	1500	1501	------	0.66 ± 0.01
13	19.67	*trans*-α-Farnesene	C_15_H_24_	1508	1507	------	25.03 ± 0.13
14	19.73	*α*-Bisabolene	C_15_H_24_	1509	1511	0.58 ± 0.03	------
15	19.84	*γ*-Cadinene	C_15_H_24_	1514	1515	1.36 ± 0.04	------
16	20.35	*α*-Sesquiphellandrene	C_15_H_24_	1516	1517	0.44 ± 0.01	------
17	20.45	*cis*-Lanceol	C_15_H_24_O	1525	1527	0.16 ± 0.02	0.09 ± 0.01
18	20.54	Isolongifolene-5-ol	C_15_H_24_O	1534	1535	0.19 ± 0.01	5.53 ± 0.07
19	20.64	Germacrene D-4-ol	C_15_H_24_	1574	1576	------	2.35 ± 0.04
20	20.81	Spathulenol	C_15_H_24_O	1576	1577	0.81 ± 0.03	0.10 ± 0.01
21	20.98	Isoaromadendrene epoxide	C_15_H_24_O	1580	1579	------	1.50 ± 0.03
22	21.41	Calarenepoxide	C_15_H_24_O	1592	1592	------	1.07 ± 0.02
23	21.56	Caryophyllene oxide	C_15_H_24_O	1594	1593	0.86 ± 0.02	0.08 ± 0.01
24	21.66	Salvial-4(14)-en-1-one	C_15_H_24_O	1595	1595	2.20 ± 0.04	0.38 ± 0.01
25	21.93	Ledene alcohol	C_15_H_24_O	1729	1731	------	0.97 ± 0.03
26	22.1	Carotol	C_15_H_26_O	1597	1598	0.78 ± 0.02	------
27	22.42	Humuladienone	C_15_H_24_O	1607	1605	------	0.32 ± 0.01
28	23.09	Neoclovenoxid	C_15_H_24_O	1608	1610	0.90 ± 0.02	0.51 ± 0.03
29	23.33	Cubenol	C_15_H_26_O	1642	1642	------	0.74 ± 0.02
30	23.49	Farnesol	C_15_H_26_O	1722	1720	1.35 ± 0.05	------
31	23.55	Ledene oxide-(i)	C_15_H_24_O	1668	1667	------	10.93 ± 0.10
32	23.65	Dendrolasin	C_15_H_22_O	1574	1575	2.85 ± 0.06	8.37 ± 0.09
33	23.81	Torreyol	C_15_H_26_O	1645	1644	------	0.55 ± 0.02
34	25	Isospathulenol	C_15_H_24_O	1625	1627	1.90 ± 0.05	------
35	25.22	tau-Muurolol	C_15_H_26_O	1646	1646	3.88 ± 0.08	0.51 ± 0.03
36	25.32	Aromadendrene oxide-2	C_15_H_24_O	1650	1649	8.81 ± 0.11	0.38 ± 0.02
37	25.85	*α*-Eudesmol	C_15_H_26_O	1652	1653	6.79 ± 0.08	0.77 ± 0.01
38	26.69	*γ*-Cadinol	C_15_H_26_O	1654	1655	0.91 ± 0.04	------
39	26.92	Dehydro-cyclolongifolene oxide	C_15_H_24_O	1657	1658	10.35 ± 0.12	------
		**Diterpenes**				**------**	**2.44%**
40	30.47	Neophytadiene	C_20_H_38_	1840	1840	------	2.44
		**Carotenoids derived compounds**				**0.28%**	**------**
41	27.36	α-Ionone	C_13_H_20_O	1426	1426	0.28 ± 0.01	------
		**Others**				**1.33%**	**0.55%**
42	18.66	1-Butanone, 1-(2,3,4,5-tetramethylphenyl)-	C_14_H_20_O	1660	1661	0.46 ± 0.03	0.32 ± 0.02
43	26.32	Methyl 2,5-octadecadiynoate	C_19_H_30_O_2_	1980	1980	0.64 ± 0.03	0.13 ± 0.01
44	42.28	*n*-Pentacosane	C_25_H_52_	2500	2500	------	0.10 ± 0.01
45	45.48	*n*-Heptacosane	C_27_H_56_	2700	2700	0.23±0.01	------
		**Total**				**96.91%**	**98.21%**

^[a]^ Rt: retention time, ^[b]^ Literature Kovats retention index on DB-5 column with reference to *n*-alkanes [36], ^[c]^ experimental Kovats retention index; values of each compound are average ± SD from duplicates. The identification of essential oil (EO) components was performed based on the (a) mass spectral data of compounds (MS) and (b) Kovats indices with those of Wiley spectral library collection and NIST (National Institute of Standards and Technology) library database.

## Data Availability

The data presented in this study are available in the article.

## Sample Availability

Samples of the compounds are not available from the authors.
